# How Mentors Think About the Attainability of Mentoring Goals: The Impact of Mentoring Type and Mentoring Context on the Anticipated Regulatory Network and Regulatory Resources of Potential Mentors for School Mentoring Programs

**DOI:** 10.3389/fpsyg.2021.737014

**Published:** 2021-11-03

**Authors:** Matthias Mader, Heidrun Stoeger, Alejandro Veas, Albert Ziegler

**Affiliations:** ^1^Department of School Research, School Development, and Evaluation, University of Regensburg, Regensburg, Germany; ^2^Department of Development Psychology and Teaching, University of Alicante, Alicante, Spain; ^3^Department of Psychology, University of Erlangen-Nuremberg, Erlangen, Germany

**Keywords:** mentors, mentoring, e-mentoring, mentor expectation, mentor training, mentoring goal, COVID-19, school mentoring

## Abstract

Research shows that trained mentors achieve better results than untrained ones. Their training should particularly address their expectations for their future mentoring. Our study involved 190 preservice teachers, potential mentors of ongoing school mentoring for primary and secondary school students of all grades. They were randomly assigned to one of four conditions in a 2-x-2 between-subjects design of mentoring type (traditional mentoring versus e-mentoring) and mentoring context (non-pandemic versus COVID-19 pandemic). Participants assessed mentoring conducted under these four conditions in terms of its appropriateness for achieving four mentoring program targets: learning, key skills, social targets, and problem coping. Participants were also asked to assess the resources available to achieve each program target. Overall, the potential mentors considered the various conditions to be suitable for achieving the four program targets. They were particularly favorable in their assessment of the possibility for the realization of learning targets. Likewise, they assumed that sufficient resources were available to achieve the targets. However, a repeated-measures MANOVA showed that the potential mentors considered more ambitious targets to be possible in traditional mentoring than in e-mentoring and normal (i.e., pre-pandemic) contextual conditions than during the COVID-19 pandemic. In contrast, they estimated the resources available to achieve the targets to be about the same in the four conditions. This indicates a decoupling of mentoring targets from the consideration of the resources needed to achieve them. This assumption was confirmed in correlation analyses and has implications for mentor training.

## Introduction

Mentoring is a context-sensitive activity in many respects. This is evident both situationally and transsituationally. Situational context specificity of mentoring means that mentor–mentee interactions are themselves shaped by the particular context in which they take place. Research on this is extensive. Examples include the mentoring format (e.g., one-on-one mentoring, group mentoring, or a hybrid form of both; Stoeger et al., 2017; National Academies of Sciences, Engineering, and Medicine, 2019; Stoeger et al., 2021b); whether mentors and mentees meet in person or, for example, have a long-distance online relationship (Knouse, 2001; Palgi and Moore, 2004; Rademaker et al., 2016); and what resources are available in mentoring (Palgi and Moore, 2004; Willems and Smet, 2007; Meyer and Bouchey, 2010; Laco and Johnson, 2019) the targets of mentor and mentee as key mentoring participants and of additional stakeholders, such as parents, friends, peers, and superiors (Meissen and Lounsbury, 1981; Young and Perrewé, 2004; Keller et al., 2018).

Transsituational context specificity means that mentoring prepares for situations that lie outside the context of the mentor–mentee interactions. For the most part, target situations take place in a different setting and without the mentors. For example, in youth mentoring, target situations may be situations that require resilience or responsible (e.g., health-related Larson) decision making (Karcher et al., 2006; Rhodes et al., 2006; Drexler et al., 2012; Karcher and Hansen, 2014). For school mentoring, target situations include, for example, improvements in studying at home and project work, dealing more effectively with bullying in school, and successful test taking (Herrera et al., 2007; Cavell and Henrie, 2010; Keller and Pryce, 2012; Schwartz et al., 2012). A mentor will generally not be on hand when a mentee is engaged in such target situations.

From a transsituational perspective, mentoring is effective to the extent to which is succeeds in preparing mentees to act competently in these target contexts. However, if target settings change, this may substantially impair the effectiveness of mentoring. The COVID-19 pandemic undoubtedly has such potential, particularly with respect to school mentoring. During the first months of 2020, governments around the world implemented various measures, such as mobility restrictions, curfews, and the closing of educational institutions (Cheng et al., 2020), to slow the spread of the novel virus. Bavarian schools and universities switched completely to online instruction and, to some extent, online exams. Distance learning under COVID-19 conditions, in turn, requires its own skill set of learning competencies (Kerres, 2020; OECD, 2020) and thus differs in many respects from in-person learning in a classroom with multiple in-person social contacts with peers and teachers, for which school mentoring typically prepares students.

However, interventions to mitigate the COVID-19 pandemic are not only changing transsituational aspects of school mentoring. Situational factors of mentoring interactions are also affected in various ways, such as with respect to the frequency of contact (Waters et al., 2002; Ayoobzadeh, 2019), the inclusion of social actors, such as parents or peers in mentoring (Keller, 2005; Keller and Blakeslee, 2014), or emotional variables (DuBois and Neville, 1997; Grossman and Rhodes, 2002; DuBois et al., 2011). In the case of emotional variables, the COVID-19 pandemic has been shown to have a particularly large impact on learners (Ahorsu et al., 2020; COSMO, 2020; National Center for Immunization and Respiratory Diseases, 2020; Zhang and Ma, 2020).

## Current Research

Our research was conducted as part of several ongoing school mentoring programs (Stoeger et al., 2021a). These take place either as e-mentoring or with in-person contact between mentees and mentors for students of all grades and tracks of primary and secondary schools in Germany. In the mentoring sessions, a wide variety of program targets is pursued, which can be assigned to four target areas: learning, social relationships (e.g., peers and mentor–mentee), key skills (e.g., persistence and assertiveness), and coping (e.g., coping with anxiety or failure). All four of these target areas have been identified in the past as areas where mentoring can improve outcomes (DuBois et al., 2011; Raposa et al., 2019).

Studies show that trained mentors are, on average, more successful at providing mentoring (Pfund et al., 2006, 2013, 2014). For this reason, training is obligatory for mentors in our school mentoring programs.^1^ Because research shows the particular importance of mentor expectations (Young and Perrewé, 2000, 2004; Kupersmidt and Rhodes, 2014; Spencer et al., 2020), we always address them in obligatory training units. This includes researching mentors’ expectations before designing a training program. The manuscript sums up the research we conducted in order to plan a mentor-training unit for implementation during the COVID-19 pandemic and the widespread remote working (for mentors) and distance learning (for mentees) it necessitated. The mentor training was designed for potential mentors (i.e., preservice teachers enrolled in undergraduate university education) for a school mentoring program. This research is conducted within the nonagonal framework of regulation in mentoring (NFR-M; Ziegler et al., 2021). The NFR-M differentiates nine regulatory dimensions that play an important role in mentoring: regulatory network, control type, regulatory function, regulatory activities, regulatory type, regulatory form, regulatory resources, and regulatory side effects.

The focus of the NFR-M is the mentoring pathway, which is defined as a sequence of mentoring episodes leading to a mentoring target. However, mentoring can include multiple targets and additional intermediate objectives, which mentors in turn adapt situationally and individually in their activities (Bloom, 1985; Kiewra, 2019). Two dimensions of the four aforementioned target areas were the focus of our preliminary studies prior to designing the mentoring training unit: regulatory network and regulatory resources.

The regulatory network dimension in the NFR-M refers to the entirety of processes that are regulated and orchestrated to reach a mentoring target. For example, mentors may differ in the relevance they ascribe to mentoring type and mentoring context when it comes to achieving the four mentoring targets (learning, social targets, key skills, and coping). Moreover, mentors may also differ on how they perceive the resources at their disposal to achieve these targets. Successful mentoring requires a variety of resources to regulate mentoring episodes. The NFR-M differentiates between endogenous resources that lie within the mentee (e.g., knowledge and motivation) and exogenous resources that lie outside the mentee, that is, in their environment (e.g., infrastructure and social support) that can help to achieve mentoring targets. These resources can be combined by mentors based on the situation of the current mentoring episode or based on advanced planning of the mentoring pathway.

The COVID-19 pandemic has the potential to influence situational as well as transsituational aspects of mentoring. We hypothesized that this should be reflected in both mentors’ perceived attainability of mentoring targets (i.e., their regulatory network) and their perceived resources to achieve mentoring targets (i.e., their regulatory resources). Specifically, we were interested in seven research questions.

Following the NFR-M (Ziegler et al., 2021), we differentiated between the dimensions of mentoring targets postulated in the model (i.e., the regulatory network) and the resources perceived for their achievement (i.e., regulatory resources). As we administered mentoring as e-mentoring and as traditional in-person mentoring, we were interested in whether mentoring type (e-mentoring versus traditional mentoring) influences perceptions of regulatory network and regulatory resources. Further, we were interested in whether the mentoring context (non-pandemic versus COVID-19 pandemic) influenced perceptions of the two dimensions. Accordingly, the first two research questions are as:

Q1: Is potential mentors’ assessment of the suitability of school mentoring for achieving mentoring targets (i.e., learning, social relationships, key skills, and coping) influenced by the mentoring type and the mentoring context?

Q2: Is potential mentors’ perception of the availability of resources for achieving mentoring targets (learning, social relationships, key skills, and coping) influenced by mentoring type and mentoring context?

The next two questions address the achievability of the four mentoring targets and the availability of resources to achieve them. Specifically, the questions are as:

Q3: In the estimation of potential mentors, which targets (learning, social relationships, key skills, and coping) can be achieved by school mentoring?

Q4: How do potential mentors perceive the resources for achieving key targets of school mentoring (learning, social relationships, key skills, and coping)?

Since mentoring type (traditional versus e-mentoring) and mentoring context (non-pandemic versus COVID-19 pandemic) may both influence the assessment of achievable targets and available resources, the next two research questions include these aspects.

Q5: In the estimation of potential mentors, which targets (learning, social relationships, key skills, and coping) can be achieved by school mentoring depending on the type (traditional versus e-mentoring) and context (normal times versus COVID-19 pandemic) of mentoring?

Q6: How do potential mentors perceive resources to achieve key targets of school mentoring (learning, social relationships, key skills, and coping) delivered either traditionally or as e-mentoring under either non-pandemic or COVID-19-pandemic conditions?

The final research question addresses a possible explanation for the decline in mentor engagement during the mentoring process to the point of dropping out (Ellison et al., 2020; Spencer et al., 2020). Ziegler et al. (2021) introduced the concept of regulatory power within the nonagonal framework of regulation in mentoring. It refers to the opportunities and resources available to mentors to achieve mentoring targets. However, in order for mentoring to be successful, the opportunities and resources must also be used competently, which Ziegler et al. referred to as regulatory insight. Therefore, it would be reasonable to assume that effective mentoring based on regulatory insight requires striving to achieve only those mentoring targets for which sufficient resources are available.

Q7: Do potential mentors take the resources into account when assessing the attainability of mentoring targets?

## Materials and Methods

The study reports findings from a larger study in which potential future mentors were asked about their expectations regarding school mentoring programs.

### Procedure

All undergraduate preservice teachers at the university of the first author were contacted who were potential mentors for one of the school mentoring programs. If they were interested in participating, they were asked to complete an online survey. By participating in the survey, preservice teachers did not commit themselves to mentoring in one of the school mentoring programs. Only 5% of the participants did have previous experience as mentors. The online survey was open 1week before and 1week after the start of the 2020 fall term.

### Participants

Of the approximately 1,200 preservice teachers for primary and secondary schools contacted, a total of 190 potential mentors participated in the study (*M*_age_=21.25; *SD*=3.53), of which 69.5% were female and 30.5% male. The sex ratio was identical in the different study conditions (*χ*^2^=0.53, *df*=1, *p*=0.46 for mentoring type; *χ*^2^=1.97, *df*=1, *p*=0.16 for mentoring context). At the end of the survey, a manipulation check was carried out in which participants were asked to indicate for which mentoring type and which mentoring context they had answered. If they answered incorrectly, they were excluded on a case-by-case basis. Informed consent was obtained from all subjects. Anonymity was assured in accordance with EU data-protection regulations.

### Study Design

Participants were randomly assigned to one of four groups based on a 2-x-2 between-subjects design with mentoring type (traditional mentoring versus e-mentoring) and mentoring context (normal times versus COVID-19 pandemic) as grouping variables. The groups differed in the introductory text that preceded the completion of the measurement instruments. The introductory text referred to either traditional mentoring or e-mentoring in non-pandemic times (i.e., “normal times”) or during the COVID-19 pandemic. The introductory text for the group of respondents being surveyed about e-mentoring during the COVID-19 pandemic was as:

*At the Department of School Research, School Development, and Evaluation, we are planning e-mentoring in which preservice teachers can participate as mentors. We would therefore like to know in advance what you think about e-mentoring during the COVID-19 pandemic*.

*In the planned e-mentoring, mentor(s) and mentee(s) will meet several times a month online and communicate about current school teaching content. Your participation will help us greatly in designing the e-mentoring during the COVID-19 pandemic*.

In the introductory text for the group of respondents being surveyed about traditional in-person mentoring, e-mentoring was replaced by mentoring, and instead of online meetings, only meetings were mentioned. In the two survey versions that did not refer to the COVID-19 pandemic, a “general assessment” of the respective mentoring type was requested instead of an assessment “during the COVID-19 pandemic.”

The study took place immediately before a second lockdown in Germany. At that time, the 7day incidence of those infected with COVID 19 exceeded the nationally defined critical level of 50 per 100,000 residents. In the city where the study was conducted, the 7day incidence even exceeded a 7day incidence of 100 newly infected persons.

### Measurement Instruments

The potential mentors were asked about their gender and age. They then completed a standardized questionnaire, which took about 25min to complete.

To measure respondents’ “regulatory networks” and “regulatory resources,” we surveyed respondents on 12 items related to four general targets of school mentoring, namely, learning (“understanding of current subject matter”, “learning skills,” and “subject performance”), social relationships (“relationship between mentor and mentee,” “teamwork skills,” and “relationship between mentee and peers”), key skills (“leadership skills,” “assertiveness,” and “perseverance”), and coping (“dealing with private problems,” “dealing with test anxiety,” and “failure coping”).

To measure respondents’ regulatory networks, potential mentors were asked to assess how well the 12 targets could be achieved in school mentoring of the respective condition. A sample item to assess respondents’ regulatory networks for e-mentoring in COVID-19 times reads, for example: “How effective is e-mentoring for supporting the following targets during the COVID-19 pandemic?” Each of the 12 targets described above were rated on a six-point Likert-type scale ranged from 1 (*not effective at all*) to 6 (*very effective*). Only the endpoints of the scales were labeled.

To measure preservice teachers’ regulatory resources, respondents were asked to assess the extent to which the resources needed to achieve the targets were available in the respective conditions of school mentoring. A sample item used to assess respondents’ regulatory resources for e-mentoring during the COVID-19 pandemic reads, for example: “In your opinion, how important is it for achieving the following e-mentoring goals that adequate resources (e.g., learning resources, learning tools, and supporting individuals) are available?” Each of the 12 mentoring targets described above were rated on a six-point Likert-type scale with the endpoints labeled “not important at all” and “very important.”

All subscales consisted of three items and were sufficiently internally consistent, with values ranging from *α*=0.67 to *α*=0.78 for respondents’ regulatory networks (learning: *α*=0.78; social relationships: α=76; key skills: *α*=0.67; and coping: *α*=0.73) and from *α*=0.69 to *α*=0.79 for regulatory resources (learning: *α*=0.79; social relationships: *α*=0.69; key skills: *α*=0.74; and coping: *α*=0.77).

### Data Analysis

In the first place, we conducted repeated-measures multivariate analyses of variance (Tabachnick and Fidell, 2007), which had mentoring type and mentoring context as independent variables and regulatory network indicators and regulatory resources as dependent variables. Analyses were performed with IBM SPSS Statistics (Version 24). Wilks Lambda was used as an appropriate estimate of *F* values for each factor. Moreover, the homogeneity of variance–covariance matrix was tested with Box’ *M* test, and the assumption of sphericity was tested with Mauchly’s test. Correlation analysis was used to explore the bivariate relations between corresponding pairs of regulatory network and regulatory resources variables.

## Results

Means and standard deviations of the dependent variables broken down by mentoring type and mentoring context can be found in Table 1. Table 2 shows the correlations. In the following, we will present the results according to the research questions.

**Table 1:** Descriptive statistics.

	Mentoring Type	E-Mentoring		Traditional Mentoring		Total	
*M*		*SD*	*M*	*SD*	*M*	*SD*
**Regulatory Network**	Learning	Normal	4.34	0.94	4.59	0.73	4.46	0.85	COVID-19	3.90	0.77	4.14	0.96	4.02	0.86
	Total	4.14	0.89	4.40	0.86	4.26	0.88
	Social relationships	Normal	3.67	1.10	4.36	0.80	4.02	1.02	COVID-19	3.28	0.93	3.35	1.22	3.31	1.07
	Total	3.49	1.04	3.92	1.12	3.70	1.10
	Key skills	Normal	3.76	0.94	4.23	0.80	3.99	0.90	COVID-19	3.40	0.72	3.59	0.94	3.49	0.83
	Total	3.60	0.86	3.95	0.91	3.77	0.90
	Coping	Normal	3.47	1.05	3.87	0.91	3.67	0.99	COVID-19	3.02	0.84	3.05	1.12	3.03	0.98
	Total	3.26	0.98	3.52	1.08	3.38	1.03
	**Regulatory Resources**									Learning	Normal	5.42	0.68	5.36	0.67	5.39	0.67	COVID-19	5.17	0.76	5.10	0.97	5.14	0.86
	Total	5.30	0.72	5.25	0.82	5.28	0.77
	Social relationships	Normal	3.99	1.11	3.85	1.12	3.92	1.11	COVID-19	4.37	1.06	4.09	0.99	4.24	1.03
	Total	4.17	1.10	3.95	1.07	4.06	1.09
Key skills	Normal	4.59	0.85	4.45	1.05	4.52	0.96	COVID-19	4.35	1.06	4.28	1.07	4.32	1.06
Total	4.48	0.95	4.38	1.06	4.43	1.01
Coping	Normal	4.66	1.10	4.28	1.11	4.47	1.11	COVID-19	4.53	1.08	4.48	1.16	4.50	1.11
Total	4.60	1.08	4.37	1.13	4.49	1.11

**Table 2:** Bivariate correlations.

	1	2	3	4	5	6	7	8
**Regulatory Network Indicators**								
1. Learning	—							
2. Social relationships	0.52^**^	—						
3. Key skills	0.54^**^	0.52^**^	—					
4. Coping	0.52^**^	0.61^**^	0.62^**^	—				
**Regulatory Resources Indicators**								
5. Learning	*0.18* ^*^	0.08	0.03	−0.02	—			
6. Social relationships	−0.15^*^	*0.03*	−0.07	0.13	0.21^**^	—		
7. Key skills	−0.12	0.06	*0.03*	0.07	0.35^**^	0.57^**^	—	
8. Coping	−0.02	0.04	−0.02	*0.17* ^*^	0.26^**^	0.58^**^	0.47^**^	—

*The correlations relevant for Q7 are set in italics*.

* *p<0.05*;

** *p<0.01*.

Considering the repeated-measures multivariate analyses of variance (MANOVA), the Box’s M test values were not statistically significant, showing homogeneity of variance–covariance matrices that were appropriate for the subsequent analyses. The MANOVA showed two statistically significant differences on the combined regulatory network indicators, *F*(1, 173)=6.91, *p*<0.01, partial *η*^2^=0.13 for mentoring type; *F*(1, 173)=26.31, *p*<0.001, partial *η*^2^=0.04 for mentoring context. The interaction of mentoring type and mentoring context did not reach the specified significance level, *F*(1, 173)=2.07, *p*=0.15, partial *η^2^*=0.01. Participants in our study thought that mentoring targets were easier to attain in traditional mentoring than in e-mentoring and under non-pandemic contextual conditions than during the COVID-19 pandemic.

The MANOVA showed no statistically significant differences on the combined regulatory resources indicators, with *F*(1, 174)=1.72, *p*=0.19, partial *η^2^*=0.01 for mentoring type; *F*(1, 174)=0.06, *p*=0.80, partial *η^2^*=0.00 for mentoring context; and *F*(1, 173)=0.06, *p*=0.80, partial *η^2^*=0.00 for the interaction of mentoring type and mentoring context. Thus, participants in our study did not differentiate in terms of perceived resources between traditional mentoring and e-mentoring or between non-pandemic times and COVID-19-pandemic times. These findings may indicate that the potential mentors focused primarily on the targets of their mentoring without thinking more deeply about the resources needed to achieve these targets.

A look at the mean scores of the scales shows that all of them are significantly above the scale mean. Thus, school mentoring is considered likely to achieve the four targets of learning, social relationships, key skills, and coping. However, the MANOVA showed mean differences between the network indicators, with *F* (3, 171)=51.70, *p*<0.001, partial *η*^2^=0.48, Wilk’s Λ=0.52. Contrasts revealed that respondents viewed learning targets as probably more achievable than the other targets, with *F*(1, 173)=59.48, *p*<0.001, partial *η*^2^=0.26; respondents deemed coping targets probably less likely to be achieved, with *F*(1, 173)=49,49, *p*<0.001, partial *η*^2^=0.35.

A look at the mean values in Table 1 shows that potential mentors are of the opinion that resources for their school mentoring are sufficiently available.

Mauchly’s test indicated that the assumption of sphericity had been violated for regulatory resources indicators, *X*^2^ (3, *N*=177)=17.04, *p*=0.004. A repeated-measures MANOVA with a Greenhouse–Geisser correction determined a statistically significant difference between regulatory resources, *F*(2.821, 490–917)=76.81, *p*<0.001, partial *η*^2^=0.31. Contrasts revealed that respondents estimated higher values for learning resources than the other resources, *F*(1, 174)=75.53, *p*<0.001, partial *η*^2^=0.30. Respondents estimated lower values for social resources than for the other resources, *F*(1, 174)=109.98, *p*<0.001, partial *η*^2^=0.39.

We calculated difference contrasts for the interaction terms from regulatory network indicators and mentoring type and mentoring context. However, no significant interactions were found (all *p*s>0.05).

We again calculated difference contrasts of the interaction terms. While no significant effects emerged for mentoring type, a counterintuitive finding emerged regarding mentoring context. While respondents assessed the availability of resources for achieving learning targets (*M*=5.39 versus *M*=5.14 for learning), key skills (*M*=4.52 versus *M*=4.31), and targets related to coping (*M*=4.4.7 versus *M*=4.51) as being more favorable or equally favorable during COVID-19-pandemic conditions in comparison with non-pandemic conditions, they rated the availability of resources for achieving social targets as being less auspicious under non-pandemic conditions than under COVID-19-pandemic conditions (*M*=3.92 versus *M*=4.24).

Potential mentors considered mentoring type and mentoring context with regard to regulatory network, but not with regard to regulatory resources. This indicates a possible disconnect between regulatory network and regulatory resources. In fact, only two corresponding correlations are statistically significant, and they are weak (see Table 2). Potential mentors who perceive more resources for learning or for coping tend to assess learning and coping targets as being more achievable.

## Discussion

Mentors’ expectations play a critical role in mentoring success (Hudson, 2013; Straus et al., 2013; Masters and Kreeger, 2017). Therefore, their expectations need to be addressed in mentor training. We do this in our school mentoring programs (Stoeger et al., 2020), which target improvements in four areas: learning, social relationships, key skills, and coping.

To design effective mentor-training units, we first screened potential mentors—preservice teachers who were university undergraduates enrolled in teacher-education programs. In a study based on the NFR-M theoretical framework (Ziegler et al., 2021) with a 2-by-2 study design with mentoring type (traditional in-person mentoring versus e-mentoring) and mentoring context (non-pandemic versus COVID-19 times) as independent variables, we asked participants how well a school mentoring program would be suited to fulfilling four mentoring targets (learning, social targets, key skills, and coping). Furthermore, we investigated how mentoring type and mentoring context influenced participants’ assessment of available resources to achieve the four mentoring targets. From the results, we hoped to gain valuable insights for designing our mentor-training programs. In the following, the most important findings will be recapitulated and their consequences for the design of our school mentoring training will be highlighted.

The first important finding is that preservice teachers assessed school mentoring as a suitable means of achieving central pedagogical targets. This is especially encouraging, because mentors in general are more successful if they can identify with the objectives of a given mentoring program (Madia and Lutz, 2004; Karcher et al., 2006; Christensen et al., 2020).

Another important finding of our study is that preservice teachers considered mentoring type and mentoring context in their assessments of the achievability of mentoring targets. In-person mentoring was rated as more suitable than e-mentoring, and non-pandemic mentoring was rated as more suitable than mentoring during the COVID-19 pandemic. In particular, the assessment of mentoring type is worth noting because, to the best of our knowledge, this is the first time such a direct comparison has been made between in-person and e-mentoring with respect to the four program targets our school mentoring programs are pursuing. However, if mentors engaged in e-mentoring should indeed—as suggested by our study participants’ assessment—exhibit lower self-efficacy, this could affect mentoring success, as research on self-efficacy suggests for both in-person mentoring (Humberd and Rouse, 2016; Varghese and Finkelstein, 2021) and counseling (Larson and Daniels, 1998). Nevertheless, as the literature does note numerous advantages for e-mentoring over in-person mentoring (Miller and Griffiths, 2005; Stoeger et al., 2021b), it would be specifically important to highlight these advantages in training for future mentors. In contrast, the more favorable assessment of the promise of school mentoring carried out when there is no pandemic seems to be an entirely realistic assessment by the participants of our study. For example, during the COVID-19 pandemic, frequency of contact (Waters et al., 2002; Ayoobzadeh, 2019), the inclusion of social actors in the mentoring (Keller, 2005; Keller and Blakeslee, 2014), or emotional variables (DuBois and Neville, 1997; Grossman and Rhodes, 2002; DuBois et al., 2011) may all be negatively affected by the circumstances arising due to the pandemic. Thus, one focus of mentor training should be on providing mentors with an accurate picture of the resources that may still be at their disposal for mentoring under specific circumstances. However, this leads us to perhaps the most surprising and, in our view, probably the most significant finding of our study.

In our study, we found several lines of evidence indicating that preservice teachers as potential mentors for school mentoring programs did not include mentoring type and mentoring context in their assessments of available resources for achieving mentoring targets. This was confirmed in both the MANOVA and correlation analyses, indicating a disconnect between assessments of the achievability of mentoring targets and the assessment of the availability of resources to achieve them. Pursuing mentoring targets without considering which resources are needed to achieve them clearly pose a risk factor for mentoring success. This disconnect of mentoring targets and available resources may also be a risk factor for the decline in mentor engagement during mentoring often observed by studies, which can result in their withdrawal from the mentoring program and thus the failure of this mentoring relationship (Ellison et al., 2020; Spencer et al., 2020).

Thus, a realistic assessment of resource–target contingency can potentially be considered an important factor in the success of mentoring (Ziegler et al., 2021). Whether this can be expected of potential mentors is questionable in light of another finding of our study, we find it counterintuitive that potential mentors considered their resources for achieving social targets as worse during non-pandemic conditions than during the COVID-19 pandemic.^2^ In our view, mentors’ perceived resource–target contingency urgently needs both further research and consideration in mentor training.

Another highly interesting finding of our research is respondents’ assessments of the targets for which mentoring is appropriate and their assessments of the availability of resources for achieving these targets with mentoring. In our view, it is not surprising that school mentoring is considered most suitable for achieving learning targets; after all, learning target is still seen as the core of school and teaching. Moreover, respondents also assessed key skills and social relationships as more promising targets than coping targets. We would like to emphasize that these assessments by the prospective mentors are neither correct nor incorrect. There is no objective standard of comparison to decide whether, for example, learning targets set and coping targets set were equally challenging and then equally well achieved in any given school mentoring program. It is interesting to note, however, that respondents’ perceptions differed with respect to the four mentoring targets. A practical consequence of our findings would be, for example, to ensure in our mentor training that mentors understand why they should strive for all program targets with the same enthusiasm. On the other hand, it also seems to be an important competence of mentors to set realistic targets in mentoring. Adequately addressing this ambiguity in the design of mentor training thus appears important in light of our findings in this study.

Similarly, resources for learning targets were rated as more favorable than for the other program targets. For achieving social program targets, on the other hand, the availability of resources was assessed the lowest. Again, there is no objective standard of comparison to decide whether potential mentors’ resource assessments are accurate in terms of the actual resources available for mentoring. Nonetheless, the differences in assessments indicate that some program targets require greater persuasion than for program targets for which resources a deemed sufficient, assuming that competent mentoring program design considers only those program targets for which sufficient resources are available (Stoeger et al., 2021b). Our results do appear to suggest that training programs should do a better job of accustoming potential mentors—especially if they have little teaching experience as was the case for the preservice teachers in our sample—to the relevance of available resources for achieving mentoring targets. Furthermore, training programs should make prospective mentors aware of additional resources for mentoring and teach them how to use these resources to achieve mentoring targets with their mentees. This stronger focus on resources in mentoring training that we propose in no way conflicts with the quite encouraging findings of our study that potential mentors are optimistic that program targets are attainable and optimistic about the availability of resources required for achieving these targets. Such training programs for mentors might benefit from using a double-loop design to address the potential mentors’ own expectations about and assessment of available resources for mentoring. After all, the lack of resource–target contingency in their assessments is too disconcerting not to specifically address in future training programs.

## Limitations and Suggestions For Future Research

We would like to conclude this article by noting a number of limitations and making suggestions for future research. Participants in our study were preservice teachers. Not all of our respondents will later serve as mentors. Thus, a survey of study participants who actually engage in mentoring after the mentor training might well have yielded somewhat different results. The same would be true for teachers already serving as mentors. They might have assessed the resources and attainability of program targets differently, based on their own experience. Future studies should therefore compare experienced with novice mentors in this respect, especially in regard to the differentiation of resources for varied mentoring contexts.

As part of the survey, the preservice teachers were asked for various assessments of things they did not know from their own experience. We had argued that this was important because mentoring success depends on mentors’ expectations (Hudson, 2013; Straus et al., 2013; Masters and Kreeger, 2017). What we do not know, however, is how easy or difficult it is to influence just these raised expectations in a mentor-training program. This would require further research.

In our study, we investigated the influence of the mentoring context, specifically the influence of the COVID-19 pandemic on mentoring. The survey was conducted during a peak of the pandemic, which may naturally affect potential mentors’ assessments. The impact of COVID-19 on subjects may diminish over time due to factors, such as gained experience, or accommodation to the new ways of remote collaboration and teaching. Such habituation to pandemic routines may eventually affect participants’ outlooks on the differences between the conditions of mentoring we investigated (traditional mentoring versus e-mentoring; normal times versus COVID-19 times). For this reason, a replication of this study during this year (or in 2022) would be helpful for drawing more robust conclusions.

In a methodological sense, and related to the previous point, a longitudinal model that includes auto-regressive path analyses will provide more objective measures for relations between variables at different time points. Moreover, as it is possible that online tools will be used more often in the future (independent of COVID-19), the reinforcement of e-mentoring could be used to increase sample size and make possible comparisons among latent constructs using structural equation modeling (SEM), reducing the measurement error. Furthermore, a bigger sample size would allow for treating the nested nature of data with multilevel SEM in addition to the analyses we performed in this study.

## Data Availability Statement

The original contributions presented in the study are included in the article/**Supplementary Material**, and further inquiries can be directed to the corresponding author.

## Ethics Statement

Ethical review and approval were not required for the study on human participants in accordance with the local legislation and institutional requirements. The patients/participants provided their written informed consent to participate in this study.

## Author Contributions

MM, AZ, and HS contributed to conception and design of the study and wrote sections of the manuscript. MM surveyed participants and organized the data. AV performed the statistical analysis. All authors contributed to manuscript revision, read, and approved the submitted version.

## Funding

This research has been funded by the Hamdan Bin Rashid Al Maktoum Foundation for Distinguished Academic Performance.

## Conflict of Interest

The authors declare that the research was conducted in the absence of any commercial or financial relationships that could be construed as a potential conflict of interest.

## Publisher’s Note

All claims expressed in this article are solely those of the authors and do not necessarily represent those of their affiliated organizations, or those of the publisher, the editors and the reviewers. Any product that may be evaluated in this article, or claim that may be made by its manufacturer, is not guaranteed or endorsed by the publisher.

## Footnotes

^1^The only exceptions are mentoring programs that are scientifically accompanied. Here, a certain number of mentors are not trained and are assigned to a control or waiting-list control group.

^2^The mentors’ reasons for this were not asked in the study. However, we asked undergraduate preservice teachers in a seminar to compare non-pandemic times with the COVID-19 pandemic in order to find out in which context social goals could be better achieved in a mentoring program. Out of 30 people who independently wrote down their assessment, none chose the COVID-19 pandemic in the direct comparison.
