# Supplementary material for: How Mentors Think About the Attainability of Mentoring Goals: The Impact of Mentoring Type and Mentoring Context on the Anticipated Regulatory Network and Regulatory Resources of Potential Mentors for School Mentoring Programs
Source: Front Psychol. 2021 Nov 3;12:737014. doi: 10.3389/fpsyg.2021.737014 (PMC8595264; doi:10.3389/fpsyg.2021.737014)
Supplement: Supplementary file 1 [file Data_Sheet_1.PDF]

Supplementary Material

1 Questionnaire

1.1 Introduction

At the Department of School Research, School Development, and Evaluation, we are planning <e-mentoring><mentoring> in which preservice teachers can participate as mentors. We would therefore like to know in advance what you think about <e-mentoring><mentoring> <during the COVID-19 pandemic><>.

In the planned e-mentoring, mentor(s) and mentee(s) will meet several times a month <in person><online> and communicate about current school teaching content. Your participation will help us greatly in designing the <e-mentoring><mentoring> <during the COVID-19 pandemic><>.

Important: There are no right and wrong answers. Therefore, even if you are not sure, we always ask you to tick an answer that you think fits best. Your participation will greatly help us in designing the <mentoring> <E-mentoring> <during the COVID-19 pandemic> on the mentees' current subject matter.

The study survey will take approximately 20 minutes to complete. Your responses will be treated completely anonymously.

1.2 Background

Gender: male / female / non-binary

Age:

Course of study: Primary school teacher / Middle school teacher / Secondary school teacher / High school teacher / Other

Subjects of study:

Have you completed an internship in a school during the 2019/2020 school year or are you currently an intern in a school?

Have you already participated in mentoring yourself?

As a mentor: yes / no

As a mentee: yes / no

1.3 Questions

1. How well can the following goals be promoted in the context of <e-mentoring><mentoring> <during the COVID-19 pandemic><>?

a. Relationship between mentor(s) and mentee
b. Understanding of current subject matter
c. Leadership skills
d. Dealing with personal problems
e. Teamwork skills
f. Learning skills
g. Assertiveness
h. Dealing with performance anxiety
i. Friendships between mentee and peers
j. Professional performance
k. Perseverance
l. Dealing with setbacks

•

2. <Mentoring><E-mentoring> differs in how many decisions the mentor makes and how many decisions mentees make independently. To what extent should mentees have autonomy in pursuing the following goals?
a. Relationship between mentor(s) and mentee
b. Understanding of current subject matter
c. Leadership skills
d. Dealing with personal problems
e. Teamwork skills
f. Learning skills
g. Assertiveness
h. Dealing with performance anxiety
i. Friendships between mentee and peers
j. Professional performance
k. Perseverance
l. Dealing with setbacks

•

3. What should be the focus of <mentoring><e-mentoring> rather with the following goals: Deepening and consolidation on the one hand or expansion and growth on the other?
a. Relationship between mentor(s) and mentee
b. Understanding of current subject matter
c. Leadership skills
d. Dealing with personal problems
e. Teamwork skills
f. Learning skills
g. Assertiveness
h. Dealing with performance anxiety
i. Friendships between mentee and peers
j. Professional performance
k. Perseverance
l. Dealing with setbacks

4. In <mentoring><e-mentoring>, sometimes the approach is more situational (that is, as the situation requires) and sometimes more planned. There are two possibilities to distinguish : *when* an activity takes place in mentoring and *what* activity takes place. In this question, we are interested in the *when*. Should mentoring activities be planned or decided situationally for the following activity areas?
   a. Relationship between mentor(s) and mentee
   b. Understanding of current subject matter
   c. Leadership skills
   d. Dealing with personal problems
   e. Teamwork skills
   f. Learning skills
   g. Assertiveness
   h. Dealing with performance anxiety
   i. Friendships between mentee and peers
   j. Professional performance
   k. Perseverance
   l. Dealing with setbacks
5. In the following, we are now interested in whether situational or planned decisions should be made about *which* activity is carried out.
   a. Relationship between mentor(s) and mentee
   b. Understanding of current subject matter
   c. Leadership skills
   d. Dealing with personal problems
   e. Teamwork skills
   f. Learning skills
   g. Assertiveness
   h. Dealing with performance anxiety
   i. Friendships between mentee and peers
   j. Professional performance
   k. Perseverance
   l. Dealing with setbacks
6. What do you think: How important is it for achieving the following goals that mentors actively gather information for their <mentoring><e-mentoring> on a regular basis (e.g., mentees' state of mind, effectiveness of support tips, current developments in mentees' lives)?
   a. Relationship between mentor(s) and mentee
   b. Understanding of current subject matter
   c. Leadership skills
   d. Dealing with personal problems
   e. Teamwork skills
   f. Learning skills
   g. Assertiveness
   h. Dealing with performance anxiety
   i. Friendships between mentee and peers
   j. Professional performance
   k. Perseverance

l. Dealing with setbacks

7. What do you think: How important is it for the achievement of the following goals that mentors actively intervene in their <mentoring><e-mentoring> in a corrective way when they see that the goals are not optimally achieved?
   a. Relationship between mentor(s) and mentee
   b. Understanding of current subject matter
   c. Leadership skills
   d. Dealing with personal problems
   e. Teamwork skills
   f. Learning skills
   g. Assertiveness
   h. Dealing with performance anxiety
   i. Friendships between mentee and peers
   j. Professional performance
   k. Perseverance
   l. Dealing with setbacks
8. How important do you think it is for achieving the following <mentoring goals><e-mentoring goals> that mentors be very proactive and preventive?
   a. Relationship between mentor(s) and mentee
   b. Understanding of current subject matter
   c. Leadership skills
   d. Dealing with personal problems
   e. Teamwork skills
   f. Learning skills
   g. Assertiveness
   h. Dealing with performance anxiety
   i. Friendships between mentee and peers
   j. Professional performance
   k. Perseverance
   l. Dealing with setbacks
9. How important do you think it is for achieving the following <mentoring goals><e-mentoring goals> that optimal boundary conditions (e.g., motivation, prior knowledge, time) prevail and that many resources (e.g., learning resources, support people) are available?
   a. Relationship between mentor(s) and mentee
   b. Understanding of current subject matter
   c. Leadership skills
   d. Dealing with personal problems
   e. Teamwork skills
   f. Learning skills
   g. Assertiveness
   h. Dealing with performance anxiety
   i. Friendships between mentee and peers
   j. Professional performance
   k. Perseverance

l. Dealing with setbacks

10. In pursuing the following <mentoring goals><e-mentoring goals>, would you also expect to see an impact on the pursuit of other <mentoring goals><e-mentoring goals> (whether positive or negative)?
    a. Relationship between mentor(s) and mentee
    b. Understanding of current subject matter
    c. Leadership skills
    d. Dealing with personal problems
    e. Teamwork skills
    f. Learning skills
    g. Assertiveness
    h. Dealing with performance anxiety
    i. Friendships between mentee and peers
    j. Professional performance
    k. Perseverance
    l. Dealing with setbacks
11. In pursuing the following <mentoring goals><e-mentoring goals>, would you also expect to see effects in the mentee's broader environment (e.g., friends, parents, classmates) that might in turn affect or promote <mentoring success><e-mentoring success>?
12. What did we just ask you about?
    a. General assessment of mentoring
    b. General assessment of e-mentoring
    c. General assessment of mentoring during the COVID-19 pandemic
    d. General assessment of e-mentoring during the COVID-19 pandemic
13. How confident are you about your statement on the survey topic?
